# PI3 kinase pathway regulated miRNome in glioblastoma: identification of miR-326 as a tumour suppressor miRNA

**DOI:** 10.1186/s12943-016-0557-8

**Published:** 2016-11-21

**Authors:** Zahid Nawaz, Vikas Patil, Yashna Paul, Alangar S. Hegde, Arimappamagan Arivazhagan, Vani Santosh, Kumaravel Somasundaram

**Affiliations:** 1 Department of Microbiology and Cell Biology, Indian Institute of Science, Bangalore, 560012 India; 2 Department of Neurosurgery, Sri Satya Sai Institute of Higher Medical Sciences, Bangalore, 560066 India; 3 Departments of Neurosurgery, National Institute of Mental Health and Neuro Sciences, Bangalore, 560029 India; 4 Departments of Neuropathology, National Institute of Mental Health and Neuro Sciences, Bangalore, 560029 India

**Keywords:** PI3 kinase pathway, LY294002, miRNome, Glioblastoma, miR-326, ARRB1, Growth suppression and RNA sequencing

## Abstract

**Background:**

Glioblastomas (GBM) continue to remain one of the most dreaded tumours that are highly infiltrative in nature and easily preclude comprehensive surgical resection. GBMs pose an intricate etiology as they are being associated with a plethora of genetic and epigenetic lesions. Misregulation of the PI3 kinase pathway is one of the most familiar events in GBM. While the PI3 kinase signalling regulated pathways and genes have been comprehensively studied, its impact on the miRNome is yet to be explored. The objective of this study was to elucidate the PI3 kinase pathway regulated miRNAs in GBM.

**Methods:**

miRNA expression profiling was conducted to monitor the differentially regulated miRNAs upon PI3 kinase pathway abrogation. qRT-PCR was used to measure the abundance of miR-326 and its host gene encoded transcript. Proliferation assay, colony suppression assay and wound healing assay were carried out in pre-miR transfected cells to investigate its role in malignant transformation. Potential targets of miR-326 were identified by transcriptome analysis of miR-326 overexpressing cells by whole RNA sequencing and selected targets were validated. Several publically available data sets were used for various investigations described above.

**Results:**

We identified several miRNA that were regulated by PI3 kinase pathway. miR-326, a GBM downregulated miRNA, was validated as one of the miRNAs whose expression was alleviated upon abrogation of the PI3 kinase pathway. Overexpression of miR-326 resulted in reduced proliferation, colony suppression and hindered the migration capacity of glioma cells. Arrestin, Beta 1 (ARRB1), the host gene of miR-326, was also downregulated in GBM and interestingly, the expression of ARRB1 was also alleviated upon inhibition of the PI3 kinase pathway, indicating similar regulation pattern. More importantly, miR-326 exhibited a significant positive correlation with ARRB1 in terms of its expression. Transcriptome analysis upon miR-326 overexpression coupled with integrative bioinformatics approach identified several putative targets of miR-326. Selected targets were validated and interestingly found to be upregulated in GBM.

**Conclusions:**

Taken together, our study uncovered the PI3 kinase regulated miRNome in GBM. miR-326, a PI3 kinase pathway inhibited miRNA, was demonstrated as a tumour suppressor miRNA in GBM.

**Electronic supplementary material:**

The online version of this article (doi:10.1186/s12943-016-0557-8) contains supplementary material, which is available to authorized users.

## Background

The last decade has seen an emergence of microRNAs (miRNAs) as the paramount biomolecules which have carved a substantial niche in almost every physiological process. A number of studies have elucidated the role of miRNAs in a wide variety of regulatory pathways which control development, differentiation, apoptosis, cell proliferation and organogenesis [1, 2]. miRNAs are a class of short, single stranded RNA molecules which do not encode for any protein, rather they modulate the expression of a myriad of proteins in a cell by fine-tuning their transcript levels. miRNAs function as guide molecules and bind to their targets with near perfect complementarity and bring about their translational repression or transcript degradation. miRNAs are synthesized from an endogenous transcript that bears a local hairpin structure, which after multiple steps of processing culminates into a 19–25 nucleotide long mature functional miRNA [3, 4]. miRNAs account for ~1 % of the genome thereby constitute one of the largest gene families [5].

miRNA biology has added an intricate complicacy in the arena of gene regulation, owing to its versatile modes of regulation. It is highly likely for a miRNA to target more than one mRNA, and similarly for one mRNA to be targeted by several miRNAs. Being epigenetic regulators of gene expression, miRNAs bring about subtle, but equally crucial changes in the expression landscape of a cell and thereby alter its phenotype. The past decade has seen a tremendous surge in the amount of literature highlighting the importance of miRNAs in malignant transformation. The aberrant expression of miRNAs has a detrimental effect on the development and progression of human cancers [6, 7]. On similar grounds, miRNAs can be categorized either as oncogenic miRNAs or tumour suppressor miRNAs depending upon their function and expression in a malignant scenario. Thus a malignant phenotype can easily be conceived to be a consequence of the misregulation of these small non-coding RNAs in a close nexus with multiple other oncogenic factors and pathways.

Astrocytomas are the most common neoplasms of the central nervous system accounting for about 60 % of the primary brain tumours in humans [8]. Glioblastoma which is a grade IV astrocytoma represents the most lethal form of intracranial tumour. The radical resection of the primary tumour mass is not curative, as glioblastomas are highly invasive and diffusely infiltrate throughout the brain. The remnant neoplastic mass which inevitably stays back after surgery, potentially leads to tumour recurrence and mortality. Glioblastomas inexorably portend grave clinical outcome and dismal prognosis, thereby rendering them the most belligerent and aggressive intracranial tumours. The current standard of care for glioblastoma management involves maximal near safe surgical resection of the tumour, followed by an aggressive adjuvant radiation therapy and chemotherapy with the oral alkylating agent temozolomide. Despite the advances in the current treatment regimens that are leveraged to manage GBM, it eludes efficient treatment as its prognosis continues to remain poor with the median survival of the patients lying at a dismal 12–15 months [9, 10]. This necessitates us to look into the intricacies of this malignancy and elucidate the novel driver events which might play an instrumental role in the initiation and progression of glioblastoma. In the wake of same, TCGA network made a concerted effort to comprehend GBM better and examined the different classes of events in GBM, like mutational, copy number alterations which affect the expression and activity of important pathways [11]. TCGA integrated large-scale multi-platform data and revealed three core pathways: *p53*, *pRb* and RTK pathways, which most often get derailed in glioblastoma. *p53* is a well-known tumour suppressor as it foils the proliferation of cells with damaged genome, by halting the cell cycle in the G1 phase or bringing about apoptosis [12]. The p53 signalling is found to be altered in 87 % of the GBM cases owing to the deletion or mutations in *p53*, *CDKN2A* and amplifications of *MDM2* and *MDM4* [11]. Similarly the *pRB* pathway, which also arrests the growth of cells by sequestering the *E2F* family of transcription factors, is found to be misregulated in 78 % of samples [11]. The third core pathway that is derailed in GBM is the Receptor Tyrosine Kinase (RTK) signalling, which is disrupted in 88 % of the GBM cases [11]. One of the key downstream signalling channels of the RTK pathway, through which the different RTKs likes *EGFR*, *ERBB2*, *PDGF* and *MET* transduce their signalling is the *phosphoinositide 3-kinase* (*PI3K*) pathway. About 15 % of the GBM samples exhibit mutations in *PI3K*. Moreover *PTEN* (*phosphatase and tensin homolog*), an enzyme which antagonizes the activity of PI3K, suffers from mutation and homozygous deletion in 36 % of GBM cases [11].

PI3 kinase pathway, which is a branch of the RTK signalling, is a pro-proliferative pathway and is drastically altered in GBM [11]. The members of the PI3 kinase pathway play a decisive role in the malignant transformation of a cell. They are actively involved in the regulation of proliferation, differentiation, migration, trafficking, and glucose homeostasis [13]. Aberrant signalling through PI3 kinase pathway results in the alteration of expression of a vast array of proteins including transcription factors which eventually mutilate the normal expression landscape of the cell. Activation of the PI3 kinase pathway is a recurrent feature observed in human tumours [14]. As the growth factor ligands bind to the membrane receptor tyrosine kinases leading to their dimerization and autophosphorylation, they trigger the activation of PI3 kinase which through their catalytic activity convert phosphatidylinositol (3,4)-bis-phosphate (PIP2) lipids to phosphatidylinositol (3,4,5)-tris-phosphate (PIP3) [15]. *PKB/Akt* binds with high affinity to PIP3 which leads to its mobilization to the plasma membrane, where it is subsequently phosphorylated on the T308 residue in its activation loop by *PDK1*, leading to its partial activation [16]. Complete activation is achieved through the phosphorylation of S473, which could be catalysed by various proteins, including *phosphoinositide-dependent kinase 2* (*PDK2*), *integrin-linked kinase* (*ILK*), *mTOR* and *DNA-dependent protein kinase* (*DNA-PK*) [17, 18]. Phosphorylated *Akt* triggers downstream pathways that regulate and support cellular growth and survival by various mechanisms, including the phosphorylation and activation of *mTOR* kinase, transcription factor *NFkB*, and *MDM2* E3 ubiquitin ligase, as well as the inactivation of pro-apoptotic protein *BAD* and *FOXO1* transcription factor, thereby aiding malignant transformation [15, 19]. In addition to the protein coding genes, the oncogenic behaviour of the *PI3K* pathway could also be attributed and extended to the deregulation of miRNAs due to its aberrant activity in the malignant state. This set of miRNAs could possibly represent a cohort of miRNAs whose levels are altered by the PI3 kinase signalling, which otherwise in untransformed astrocytes regulate and fine-tune the levels of various oncogenes including RTKs.

In the current study, we identified a cohort of miRNAs that are regulated by PI3 kinase signalling. We further shortlisted and validated miR-326, an intragenic miRNA, as a downregulated miRNA in GBM, whose levels are brought down by PI3 kinase activity. Additionally, we also observed that the host gene of miR-326, ARRB1 is also subjected to similar regulation by the PI3 kinase signalling.

## Methods

### Human tumour samples

In this study, the glioblastoma tissue samples that were used were obtained from the patients who were operated in Sri Sathya Sai Institute of Higher Medical Sciences (SSSIHMS) and National Institute of Mental Health and Neurosciences (NIMHANS), Bangalore, India. We used non-tumour brain which comprised of anterior temporal lobe of the brain for the purpose of comparison. The non-tumour brain was obtained during the surgery of intractable epilepsy patients. The tissues both tumour and non-tumour control were snap-frozen in liquid nitrogen and stored at −80 °C and used for RNA isolation. A total of 56 GBM samples and 14 control brain samples were used in this study. The different experimental protocols methods and adopted in this study strictly follow the guidelines approved by the Institutional biosafety clearance committee of Indian Institute of Science, Bangalore.

### Cell culture

Glioma cell lines U87, U138, U251, U343, U373, LN229, LN18 and T98G were grown in Dulbecco’s Modified Eagle’s medium (DMEM, Sigma) while SVG cells were grown in MEM. The medium was supplemented with 10 % foetal bovine serum (FBS, Gibco, ThermoFisher) along with penicillin and streptomycin. The cells were maintained in in a humidified incubator at 37 °C and 5 % CO_2_. Most of the used cell lines were procured from ECACC (European Collection of Authenticated Cell Cultures). Immortalized human astrocytes (IHA) were a kind gift from late Dr. Abhijit Guha.

### RNA extraction, cDNA conversion and quantitative RT-PCR analysis

Total cellular RNA including the miRNA fraction was isolated using Trizol reagent (sigma). RNA thus isolated was analysed for its purity and integrity by Nano-drop and gel electrophoresis. Two μg of total RNA were used for cDNA conversion using High capacity cDNA reverse transcription kit the (Applied Biosystems, USA) according to the manufacturer’s protocol. cDNA generated was diluted with nuclease free water in the ratio of 1: 10 such that the final concentration of the cDNA was 10 ng/μl. Real-time PCR was done using the ABI PRISM 7900 HT Sequence Detection System (Life technologies, USA) under default conditions: 95 °C for 15 min, 40 cycles of 95 °C for 15 s, 60 °C for 20 s and 72 °C for 25 s. Expression was analyzed using GAPDH, 18S RNA and RPL35a as a reference gene and the ddCt method [20].

### Real-Time qPCR for quantification of miRNAs

For the detection and estimation of miRNAs, we employed a TaqMan based real-time PCR method (Applied Biosystems). The TaqMan real-time PCR method involves the formation of miRNA-specific cDNA from total RNA (10 ng) using a specific stem-loop primer. A diluted reverse transcription product, which is a miRNA specific cDNA was used for each real-time PCR reaction. Real-time PCR was performed using the ABI PRISM 7900 HT Sequence Detection System (Life technologies, USA). The thermal profile of the PCR was as follows: 95 °C for 10 min and then 40 cycles of 95 °C for 15 s and 60 °C for 1 min. U6, U44 and U48 small RNAs, in different combinations, were used as endogenous control. ΔΔC_T_ method was used for the calculation of expression ratios.

### Pre-miR mimic transfection of glioma cells

In order to overexpress the down regulated miRNAs, the precursors or mimics of corresponding miRNAs were transfected into the glioma cell lines. The pre-miR mimics and Cy-3- labelled Pre-miR control/unlabelled pre-miR negative controls were obtained from *Ambion*. The transfection of pre-miRs and their respective negative controls involve a method called reverse transfection wherein the transfection mix is added first to an empty dish and then the dish is overlaid with the requisite volume of cell suspension. The method involved the dilution of siPORT transfection reagent (Ambion) in a requisite volume of OptiMEM medium (reduced serum medium) and this mix was incubated at room temperature for 10 min. Meanwhile the pre-miRs and their respective pre-miR-negative controls (10 μM stock) were also diluted at a particular concentration in OptiMEM. After 10 min of incubation the diluted siPORT and the diluted pre-miR were mixed together and again incubated at room temperature for 10 more minutes. The mix was finally added to an empty dish wherein the cell suspension was overlaid subsequently at a desired cell density. The dish was incubated in CO_2_ incubator. RNA was isolated at the indicated time points and the overexpression of miRNA was confirmed by assaying for the level of mature miRNA by TaqMan quantitative real-time PCR as explained before.

### Colony suppression assay

Colony forming ability of cells upon miR overexpression was gauged by initial transfection of negative control pre-miR or pre-miR-326 in glioma cells. After 36 h of transfection, cells were counted in each group and 2500 or 5000 cells were plated in a 12 well cluster plate. The culture media was replaced every 3 days. After 2–3 weeks of plating, the colonies were fixed in chilled methanol for 30 min and stained with 0.05 % crystal violet for another 30 min and photographed.

### Proliferation assay

Cells were transfected with negative control pre-miR or pre-miRs for miR-326. After 24 h, cells were harvested and plated (500 cells per well) in triplicates in a 96-well cluster plate. Each day a triplicate set in each condition was treated with MTT to quantify the number of viable cells. 20 μl of MTT (5 mg/ml in PBS) was added to the cells in 96-well plate. Three hours after MTT addition, the formazan crystals were dissolved in DMSO (200 μl) and measured as absorbance at 570 nm.

### Wound healing scratch assay

Cells were initially transfected with negative control pre-miR or pre-miR-326 and after 24 h of transfection they were seeded in a 12 well cluster plate at a high density. After 12–24 h, the monolayers were mechanically disrupted with a sterile toothpick to produce a clean uniform scratch. The assay was performed in duplicates. The wells were photographed every 12 h and the gap distances were recorded at each time point to assess the closure of the wound.

### Western blotting

Western blotting was carried out to assay the changes in protein expression or modification. Cells were washed with chilled PBS and harvested by trypsinization. Cells were lysed using RIPA (RadioImmunoPrecipitation Assay) buffer. Requisite volume of RIPA buffer was added to the cell pellet and the cell suspension was vigorously agitated and vortexed and lastly incubated on ice for 30 min to allow cell lysis. This was followed by centrifugation at 14,000 rpm for 15 min at 4 °C. The clear supernatant or the protein lysate was collected in a fresh centrifuge tube and stored at −20 °C. Protein lysates were quantified by Bradford assay reagent (Bio-Rad) before subjecting the same to immunoblotting. The protein were separated on 10 or 12 % SDS-PAGE and then transferred to PVDF (Polyvinylidene difluoride) membrane (Merck Millipore). Samples belonging to a particular experiment were run in a same gel under same experimental conditions. The membrane was probed with the required antibody at a prescribed dilution overnight at 4 °C with gentle rocking. The antibodies that were used in the study include: Phospho-Akt (Ser473) (Cell Signalling technology; cat # 4060), Akt (Cell Signalling technology; cat # 9272), α-Tubulin (Mouse mAb (DM1A); cat # CP06).

### miRNA microarray and data normalization

Total RNA (1 μg) from LY294002 treated and DMSO control was labelled with Hy3 and Hy5 fluorescent label, respectively, using the miRCURY LNA Array power labelling kit (Exiqon, Denmark). The common reference RNA was prepared by mixing equal quantity of RNA from all the samples. The Hy3-labeled samples and Hy5-labeled reference RNA sample were mixed pair wise and hybridized to the miRCURY LNA™ microRNA Array (6th Gen) (Exiqon). The array has capture probes for all the microRNAs in human, mouse, rat and their related viruses as annotated in miRBase Release v16.0. The capture probes are Locked Nucleic Acid (LNA™) enhanced oligonucleotides, complementary to mature microRNAs. The experiment involves a competitive hybridization of each sample with a reference pool. The hybridization was performed using a Tecan HS4800 hybridization station (Tecan, Austria). The miRCURY LNA Array microarray slides were scanned using the G2565BA Microarray Scanner System (Agilent, USA) and image analysis was performed using the ImaGene 7.0 software (BioDiscovery, USA). The quantified signals were background-corrected (Normexp with offset value 10) and normalized using the global Lowess (LOcally WEighted Scatterplot Smoothing) regression algorithm. The median ratio of Hy3/Hy5 intensity for replicate spots for each miRNA was obtained after normalization. The values for Hy3/Hy5 ratio were log_2_-transformed. The difference in log_2_ ratio for each miRNA (subtracting the log_2_ ratio of a given sample from its respective control sample) was considered in subsequent analyses.

### Transcriptome analysis by RNA sequencing

The RNA samples were assessed for quality and quantity using Agilent’s Bioanalyser. 1 ug of RNA from each sample was used for library preparation. Each sample was run in duplicates to obtain the data. The library for sequencing was prepared using TrueSeq RNA sample preparation kit as per the manufacturer’s guidelines (Cat # RS-122- 2001). The library was then re-quantified using Agilent’s Bioanalyser as well as real-time qPCR. 120 ul (10pM) of each sample library were taken, the strands were denatured and finally subjected to cluster generation on the flow-cell in the c-Bot system using TruSeq PE Cluster kit (Cat # PE-401-3001). The flow cell was finally subjected to two rounds of sequencing (Read1 and Read2) and the results were obtained as intensity files. Sequencing was conducted on Illumina HiScanSQ using Truseq SBS V3 technology for 50 base pair paired-end reads RNA sequencing (Cat # FC-401-3002). Raw reads obtained were mapped to the human reference genome (hg19) using TopHat (version 2.0.10). The alignment files were thereafter subjected to Cufflink (version 2.2.0) to generate a transcriptome assembly. Each of the transcriptome assemblies was merged using Cuffmerge utility, to generate the final transcriptome assembly. The transcriptome assembly thereby generated and alignment file were analysed through Cuffquant utility to quantify gene and transcript expression. Normalization of quantified gene and transcript expression was carried out using Cuffnorm utility. Differentially expressed genes were identified using Cuffdiff utility and those having fold change above 1.5 with FDR-adjusted *p*-value <0.05 were considered significant.

### Transcription factor (TF) analysis

A 6 kb region in the promoter of ARRB1 that was marked by significant enrichment of histone activation marks was analysed for TF analysis. We used Matinspector, an online transcription factor analysis tool to identify the putative transcription factors that could potentially bind and regulate the expression of ARRB1 locus. The transcription factors identified by Matinspector were further analysed for their regulatory dependence on the PI3 kinase pathway. To elucidate the transcription factors that were regulated by PI3 kinase pathway, we analysed the expression of these TFs in two sample cohorts characterised by high and low phospho-Akt levels. Essentially, we divided the TCGA GBM samples from the Reverse Phase Protein Array (RPPA) into four quartiles based on their phospho-Akt ser-473 levels (https://cancergenome.nih.gov/). The samples that formed the two extreme quartiles were considered as high phospho-Akt samples (top 25 %, *n* = 49) and low phospho-Akt samples (bottom 25 %, *n* = 49). The differential expression of the TFs (output from Matinspector) was finally compared only in these two sample cohorts to assess their PI3 kinase dependent regulation.

### Histone landscape analysis

The histone marks across the ARRB1 locus were analysed from the “Integrated Regulation from ENCODE Tracks”. The landscape gave a picture of H3K4Me1 (found near regulatory elements), H3K4Me3 (found near promoters) and H3K27Ac (found near active regulatory elements). The information about these histone marks was derived from 7 cell lines from ENCODE. It also provided the transcript levels assayed by RNA-seq on 9 cell lines from ENCODE.

(https://genome.ucsc.edu/cgi-bin/hgTrackUi?db=hg19&g=wgEncodeReg).

### Other data sets

In order to compare and corroborate the expression of miR-326 and other genes evaluated in the study, the fold change was further derived from various publicly available data portals, including The Cancer Genome Atlas (TCGA) dataset (Agilent and Affymetrix platforms), REMBRANDT dataset and GSE22867 dataset. TCGA Agilent and Affymetrix data was accessed and downloaded on 24/1/2013 (http://cancergenome.nih.gov/). The REMBRANDT data was accessed and downloaded on 17/2/2011 (ftp://caftpd.nci.nih.gov/pub/caARRAY/experiments/caArray_fine-00037/). GSE22866 data was accessed and downloaded on 08/07/2013.

### Bioinformatic analysis

Gene targets for miR-326 were predicted using miRWalk (http://zmf.umm.uni-heidelberg.de/apps/zmf/mirwalk2/), an online target prediction tool which provides results compiled from 12 algorithms.

### Gene ontology and pathway analysis

Gene ontology mining and pathway analysis was carried out using DAVID (https://david.ncifcrf.gov/) Bioinformatics Resources tool.

### Statistical analysis

Statistical significance between two groups was calculated by unpaired student’s *t*-test using the GraphPad PRISM software. In experiments involving more than one group for comparison, ANOVA was used with a suitable post-hoc test. In the correlation study, we used spearman correlation method to calculate the correlation coefficient. The statistical significance is indicated as asterisks (*). *P* < 0.05 was considered to be statistically significant, (ns) not significant; (*) *p* ≤ 0.05; (**) *p* ≤ 0.01 and (***) *p* ≤ 0.001.

## Results

### miRNAs regulated by PI3 Kinase pathway

**Fig. 1:**
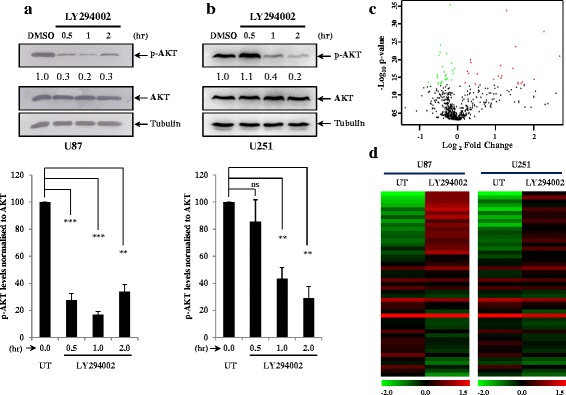
PI3 kinase pathway regulated miRNAs in glioblastoma **a**, **b**. Western blot data indicating the time course loss of phosphorylation of AKT upon PI3 kinase inhibition by LY294002 (50 μM) treatment in two different cell lines U87, and U251 respectively. Also indicated is the densitometric quantification of p-AKT/AKT levels at different time points. The first bar of each plot represents the untreated (UT) condition, where equal volume of DMSO was added as vehicle control. The quantitation and the statistics in the column plots has been deduced from three independent experiments. *p* values were calculated by student’s *t*-test and the symbols are indicated. (**) *p* ≤ 0.01; (***) *p* ≤ 0.001. **c**. Volcano plot representing the significantly regulated miRNAs upon LY294002 treatment, 15 miRNAs (indicated by red dots) were found to be upregulated upon PI3 kinase pathway inhibition and 1 (indicated by green dots) miRNA was upregulated. miRNAs were considered significant based on their *p value* and differential Log_2_ ratio (more than +/− 0.57 fold). **d**. Heat map of the 47 miRNAs derived from the volcano plot which were significantly different upon LY294002 treatment in U87 and U251 cell lines

PI3 kinase pathway is one of the most genetically altered pathways in glioblastoma and thereby potentiates the aberrant expression of a number of oncogenes and tumour suppressor genes downstream to it. With an objective to identify the miRNAs regulated by the activated PI3 kinase pathway in GBM, we examined the expression of miRNAs after abrogating the pathway in U87 and U251 glioma cell lines. Cells were treated with LY294002, a selective, potent inhibitor of PI3 kinase which essentially inhibits the activity of phosphatidylinositol-4,5-bisphosphate 3-kinase and thereby the synthesis of phosphatidylinositol (3,4,5)-trisphosphate. This inhibition eventually blocks the phosphorylation of Akt (PKB) and the subsequent signaling. We probed the activity of PI3 kinase inhibition after LY294002 treatment by reading out the phosphorylation status of its target protein Akt through western blot analysis. We demonstrated that LY294002 inhibited the PI3 kinase pathway as early as 30 min, as the levels of phospho- Akt were significantly reduced at least till two hours of treatment in U87 cell line. In U251 cell line, the phosphorylation of Akt was lost at 60 min of treatment but continued to remain less till two hours of examination (Fig. 1a and b).

To unveil the miRNAs regulated by the PI3 kinase pathway, we isolated RNA after 24 h of LY294002 treatment from the two different glioma cell lines: U87 and U25, and subjected it to miRNA micro-array using miRCURY LNA™ microRNA Array (6th Gen) platform developed by Exiqon Inc. We observed a large number of miRNAs being differentially expressed upon PI3 kinase pathway inhibition in both U87 and U251 cell lines as indicated in the volcano plot (Fig. 1c). There were 22 upregulated miRNAs and 25 downregulated miRNAs that were significantly different between the untreated and LY294002 treatment condition (Additional file 1: Table S1). The differential regulation of these miRNAs in each of the cell lines (U87 and U251) is shown in the form of a heat map (Fig. 1d). By applying an additional cut-off of +/− 0.57 fold change (Log_2_ scale), we were able to narrow down to 16 unique miRNAs that were commonly and similarly regulated in U87 and U251 cells (Additional file 2: Table S2). Next, we investigated the expression of these miRNA in GBM tumour samples. We hypothesized that if a miRNA gets upregulated upon LY294004 treatment, it must be downregulated in GBM and vice versa. From this analysis, we inferred that if a particular miRNA is repressed in GBM by an activated PI3 kinase pathway, the inhibition of pathway would result in alleviating the levels of that miRNA, resulting in its upregulation. Thus, we relied upon the differing expression of miRNAs between LY294002 treated cells and their expression in GBM (Additional file 2: Table S2). Based on the limited miRNA expression data available, we were able to identify three miRNAs: miR-326, miR483-5p and miRPlus-C1115 which were downregulated in GBM but the inhibition of the PI3 kinase pathway alleviated their transcript levels (Additional file 2: Table S2).

### miR-326 is downregulated by the PI3 kinase activity

**Fig. 2:**
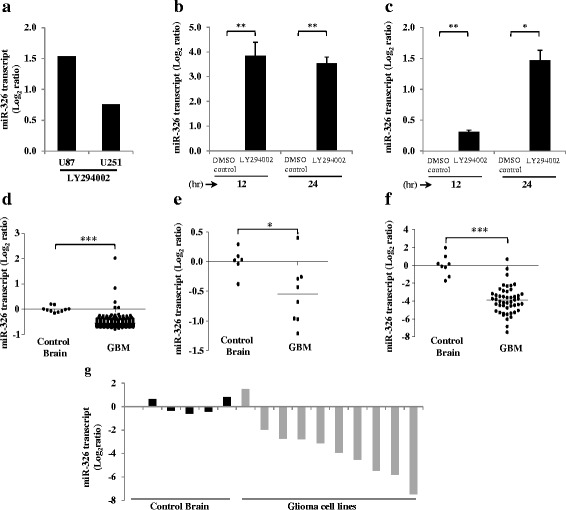
miR-326 is a PI3 kinase regulated miRNA and is downregulated in glioblastoma. **a**. Transcript levels of miR-326 in U87 and U251 cells upon 24 h of LY294002 treatment in the miRNA-microarray. **b**, **c**. Transcript levels of miR-326 in U87 and U251 cells upon PI3 kinase pathway inhibition by LY294002 treatment (50 μM). The transcript levels were assayed at 12 and 24 h time points by real time quantitative PCR. **d**, Scatter plot depicting the downregulation of miR-326 in GBM in the TCGA dataset as compared to control brain samples. **e**, Scatter plot depicting the downregulation of miR-326 in GBM in the lab dataset (microarray), each dot represents one sample and for each tumour sample fold change is calculated as difference in the expression value of the miRNA over the average of its expression values in the control brain samples. **f**, Scatter plot depicting the Log_2_ transformed miR-326 expression values obtained from qRT-PCR analysis in an independent cohort of GBM samples as compared to control brain samples. **g**, Log_2_ transformed miR-326 expression values obtained from qRT-PCR analysis in GBM cell lines LN18, U373, T98G, LN229, U343, SVG, IHA, U138, U251 and U87. For each GBM sample or cell line, fold change in miRNA expression is calculated over its mean expression in control brain samples. *p* values were calculated by student’s *t*-test and the symbols are indicated. (*) *p* ≤ 0.05; (**) *p* ≤ 0.01; (***) *p* ≤ 0.001

From the microarray data, we found that miR-326 was one of the upregulated miRNAs upon PI3 kinase pathway inhibition in both U87 and U251 cell lines (Fig. 2a). We further validated our array results by quantitative PCR, which revealed the upregulation of miR-326 upon LY294002 treatment at two different time points in U87 and U251 cell lines by qRT-PCR (Fig. 2b and c). Being upregulated upon inhibition of the PI3 kinase pathway, we expected miR-326 to be downregulated in GBM. In fact, we observed that miR-326 was significantly downregulated in GBM compared to control brain in the TCGA data set as well as in our lab microarray dataset (Fig. 2d and e) (Additional file 3: Table S3a, b). We also assayed the levels of miR-326 in an independent sample cohort using qRT-PCR and demonstrated that indeed miR-326 was a down-regulated miRNA in GBM (Fig. 2f) (Additional file 3: Table S3c). As expected the expression levels of miR-326 were also significantly low in most of the glioma cell lines we examined - SVG, IHA, LN18, U373, T98G, LN229, U343, U138, U251 and U87 (Fig. 2g). Thus from our results, we conclude that miR-326 is a downregulated miRNA in GBM and its low expression is brought about by aberrant PI3 kinase signaling.

### miR-326 over-expression exerts tumour suppressive effects in glioblastoma

**Fig. 3:**
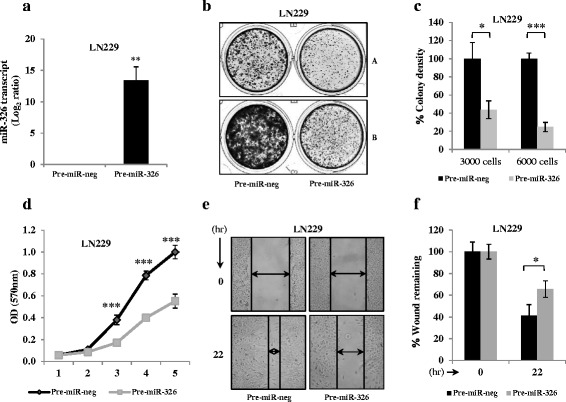
miR-326 over-expression exerts tumour suppressive effects in glioblastoma. **a**. Log_2_ transformed miR-326 expression value obtained from qRT-PCR analysis upon its over-expression in LN229 cell line. **b**, **c**. LN229 cells were transfected with Pre-miR-neg or Pre-miR-326, cells were counted and indicated numbers of cells (**a** = 3000 and **b** = 6000 cells) were plated in duplicates in a 12-well cluster plate. After 3 weeks of plating, the colonies were stained with crystal violet, photographed (**b**) and counted (**c**). % colony density and ± Sd is plotted. **d**. LN229 cells transfected with Pre-miR-neg or Pre-miR-326 were subjected to proliferation assay. Proliferation was assessed by MTT assay, measuring OD (570 nm) for both miR-neg and miR-326 at each time point. For each condition and time point, the samples were plated in triplicates and the standard deviation was calculated over three independent biological replicates. Significance is indicated for different time points. **e**, **f**. LN229 cells transfected with Pre-miR-neg or Pre-miR-326 were subjected to scratch assay (**e**), migratory potential of cells was quantitated by the percent of wound remaining after the indicated time point (**f**). *p* values were calculated by student’s *t*-test using graph pad prism and the symbols are indicated. (*) *p* ≤ 0.05; (**) *p* ≤ 0.01; (***) *p* ≤ 0.001

Since miR-326 is downregulated by the activated PI3 kinase pathway and is lowly expressed in GBM, we over-expressed it by pre-miR-326 transfection in LN229 cell line and studied its effect on various parameters related to cancer phenotype. The pre-miR-326 transfection indeed resulted in high levels of mature miR-326 in LN229 cells (Fig. 3a). We observed that the re-expression of miR-326 induced significant reduction in colony forming ability of glioma cells as well as inhibited the rate of proliferation (Fig. 3b, c and d). In addition to that miR-326 over-expression also retarded the migration of cells to fill the gap, mimicking a wound (Fig. 3e and f). These data indicate that miR-326 being down-regulated in GBM has tumour suppressive effects as it imparts colony suppression and inhibits the proliferation of cells as well as their migratory potential.

### ARRB1, the host gene of miR-326 is also regulated by PI3 Kinase pathway

**Fig. 4:**
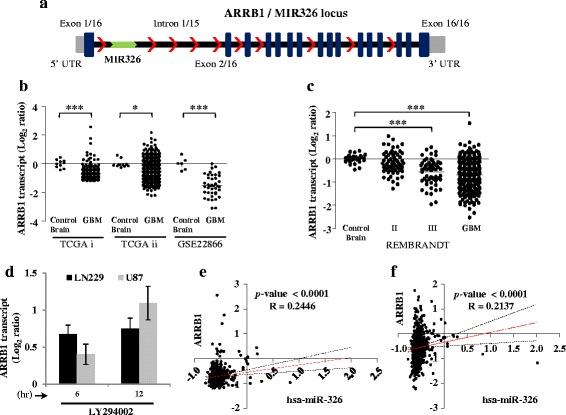
Arrestin beta 1, the host gene of miR-326 is also regulated by PI3 kinase pathway and is downregulated in GBM. **a**. Schematic representation of the genomic locus of MIR326 gene in the intronic region of ARRB1 gene. MIR326 resides in the first intron of ARRB1. **b**, **c**. Log_2_ transformed ARRB1 expression values obtained from different publicly available expression data sets, TCGA i (Affymetrix), TCGA ii (Agilent), GSE22866 and REMBRANDT data set respectively. Each dot represents one sample and for each sample fold change in expression is calculated over its mean expression in control brain samples. *p* values were calculated by student’s *t*-test or ANOVA (**c**) and the symbols are indicated. (*) *p* ≤ 0.05; (***) *p* ≤ 0.001. **d**, **e**. Correlation graph between hsa-miR-326 and ARRB1 transcripts using two different expression platforms from TCGA agilent (**d**) and affymetrix (**e**). ARRB1 and miR-326 exhibit a significant positive correlation in their abundance with a correlation coefficient *R* = 0.2446 and *p* < 0.0001 (agilent) and *R* = 0.2137 and *p* < 0.0001 (Affymetrix). **f**. Log_2_ transformed expression values of ARRB1 upon PI3 kinase pathway inhibition with LY294002 treatment (50 μM) in two different cell lines LN229 and U87 for two time points (6, 12 h)

miR-326 is an intragenic miRNA and is located in the first intron of the gene Arrestin Beta-1 (ARRB1) (Fig. 4a). Since miR-326 was regulated by the PI3 kinase pathway, we hypothesized whether its host gene, ARRB1 is also subjected to PI3 kinase signaling mediated regulation. We initially analysed the transcript levels of ARRB1 in GBM across different publicly available data sets and observed that it was significantly down-regulated in GBM as well as in the lower grades (Fig. 4b and c) (Additional file 3: Table S3d, e, f, g). Further, to determine whether ARRB1 was regulated by the PI3 kinase pathway, we measured its expression upon PI3 kinase inhibition in two cell lines: LN229 and U87. We observed that indeed like miR-326, the transcript level of ARRB1 was also getting alleviated, indicating that ARRB1 was also negatively regulated by the PI3 kinase signalling pathway (Fig. 4d). This observation led us to the assumption that possibly miR-326 and ARRB1 are under the control of a common promoter upstream to ARRB1 locus. Next, to substantiate our assumption, we analysed the expression of ARRB1 and miR-326 in an identical sample set to figure out if there exists any close relation in terms of expression, between the two. We used ARRB1 transcript levels from two different platforms (Affymetrix and Agilent) of the TCGA dataset and correlated them with miR-326 expression in an identical sample cohort. We found that there exists a significant positive correlation between miR-326 and ARRB1 transcripts in GBM (Fig. 4e and f). However, the correlation coefficient was not so high, which suggested the possibility that ARRB1 and miR-326 transcripts might have differing relative stabilities and half-lives. In addition, it is also possible that miR-326 may be transcribed from its own independent promoter. In order to investigate this possibility, we analysed the read counts corresponding to various introns and exons of the ARRB1 gene derived from the RNA sequencing data from TCGA. Assuming that ARRB1 and miR-326 are transcribed from independent promoters, we expected to observe higher number of transcript reads corresponding to intron-1 as against the remaining introns of the ARRB1 gene. On the contrary, we observed that the normalized read count of the region corresponding to intron-1 was no different from the remaining introns of ARRB1 gene in control brain and GBM tissue samples (Additional file 4: Figure S1a, b), strongly suggesting that this intron did not give rise to any primary miRNA corresponding to miR-326 independently. Moreover, the normalised read count of the intron-1 was significantly less than that of exon-1, further consolidating our hypothesis that miR-326 transcript was not synthesized independent of its host gene ARRB1. Additionally, we also evaluated the active histone marks across the ARRB1 locus using “Integrated regulation from ENCODE tracks”. While only one of the histone mark H3K4Me1 which is essentially suggestive of enhancer region, was enriched in the intronic region upstream to miR-326 locus, all the three marks H3K4Me1, H3K4Me3 and H3K27Ac were enriched in the ARRB1 promoter region (Additional file 4: Figure S1c). Importantly, the enrichment of H3K4Me3 mark was worth noticing in the region upstream to ARRB1, as this histone mark is characteristic of promoters. This data provides yet another evidence that the PI3 kinase pathway essentially regulates ARRB1 promoter, thereby modulating the expression of miR-326 as well as ARRB1.

Next, to ascertain the mechanism through which PI3 kinase pathway brought about this regulation, we examined the transcription factors that could putatively bind to the promoter region of ARRB1 (see Methods for detail). A segment of 6 Kb in length in the region covering the active histone activation marks towards the 5’ end of ARRB1 was analysed for transcription factor binding using Matinspector, an online transcription factor analysis tool. Since ARRB1 and miR-326 are co-regulated by the PI3 kinase pathway, we expected the transcription factors controlling their expression also to be regulated by the PI3 kinase pathway. We divided the samples from the TCGA Reverse Phase Protein Array (RPPA) data between high and low phospho-Akt groups to identify the differentially regulated transcription factors. A list of 1831 transcription factor (TF) entries obtained from Matinspector TF analysis, were assayed for their expression levels in GBM between high and low activated Akt TCGA samples (Additional file 4: Figure S2). We obtained 48 TF entries that had significantly different expression levels between high and low Akt samples, out of which 22 TF entries had more expression in low Akt GBM samples which could potentially be the transcriptional activators of ARRB1 and are suppressed by the PI3 kinase pathway (Additional file 4: Figure S2). We also obtained 26 TF entries that had more expression in high Akt GBM samples which could potentially be transcriptional repressors of ARRB1 and are activated by the PI3 kinase pathway (Additional file 4: Figure S2). To further narrow down on the potential transcription factors that regulated ARRB1 locus, we chose those TFs that had a binding site on the negative DNA strand as both miR-326 and ARRB1 reside on the negative strand (Additional file 5: Table S4). We shortlisted 4 unique potential transcriptional activators and 7 potential transcriptional repressors, as many of the TF entries had more than one binding site and finally subjected them to correlation analysis with ARRB1 transcript levels (Additional file 4: Figure S2). Out of 4 transcription activators, Zic family member 3 (ZIC3) alone significantly and positively correlated with ARRB1 expression (Table 1), while as out of 7 transcription repressors SRY-box 6 (SOX6), INSM transcriptional repressor 1 (INSM1) and zinc finger protein 300 (ZNF300) significantly and negatively correlated with ARRB1 expression (Table 1). Thus, it appears PI3 kinase regulates these transcription factors ZIC3, SOX6, INSM1 and ZNF300 which eventually results in the downregulation of ARRB1 and miR-326 in GBM.

**Table 1:** PI3 kinase regulated transcription factors that can impact the expression of ARRB1 genetic locus

S.No	Gene	Log_2_ FC p-Akt high vs low	No of binding sites	Regulation in GBM Log_2_ fold change	Correlation with ARRB1 (R)	*p*-value
1	ZIC3	−0.53	6	−0.35 (Down)	+0.085	0.0408
3	SOX6	0.50	4	0.57 (Up)	−0.125	0.0025
4	INSM1	0.50	2	0.69 (Up)	−0.141	0.0006
5	ZNF300	0.41	1	1.69 (Up)	−0.230	<0.0001

### miR-326 regulated genes demonstrate its role in gliomagenesis

**Fig. 5:**
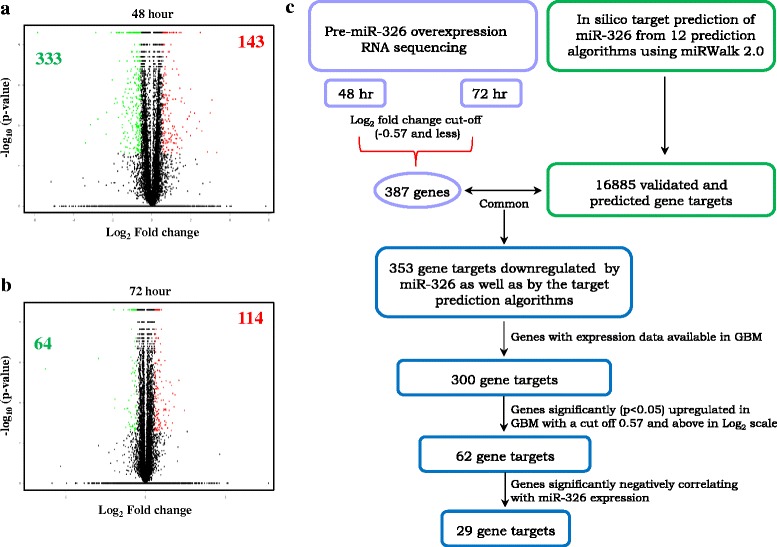
Transcriptome analysis upon miR-326 overexpression. **a**, **b**. Volcano plot representation of the RNA sequencing data, indicating the genes significantly and differentially expressed between Pre-miR-neg and Pre-miR-326 condition in LN229 cell line at two 48 h and 72 h time points respectively. Each red dot indicates a gene significantly upregulated in Pre-miR-326 overexpression condition and each green dot indicates a gene significantly downregulated in Pre-miR-326 overexpression condition. The figures (bolded) inside each plot denote the number of genes upregulated and downregulated significantly, Green indicates the downregulated genes and red indicates the upregulated genes. **c**. Schematic representation of miRNA target prediction. All the genes downregulated (−0.57 and less in the Log_2_ scale) upon miR-326 overexpression from both 48 and 72 h time point were sought for the analysis. miR-326 targets were derived from 12 target prediction algorithms (miRWalk, MicroT4, miRanda, miRBridge, miRDB, miRMap, miRNAMap, PICTAR2, PITA, RNA22, RNAhybrid, Targetscan) assembled in miRWalk 2.0 online tool. The experimental targets were compared with the *in silico* targets to find out the common ones, which were finally compared for their expression in GBM and their correlation with miR-326 expression

**Fig. 6:**
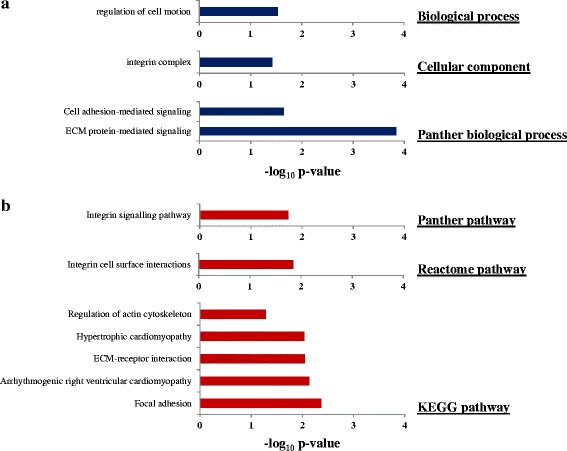
Gene ontology analysis of the putative targets of miR-326. **a**, **b**. DAVID based gene ontology analysis (**a**) and pathway analysis (**b**) of the 29 putative targets of miR-326 indicates significant enrichment of the cell migration and invasion related terms and pathways

In order to find the putative downstream targets (both direct and indirect) of miR-326 in GBM, we overexpressed miR-326 by pre-miR transfection and carried out the transcriptome analysis by RNA sequencing at two different time points (48 and 72 h) in LN229 cell line. We observed a multitude of genes getting regulated after miR-326 overexpression at both time points as depicted through the volcano plots (Fig. 5a and b). We shortlisted the genes regulated by miR-326 based on their fold change and *p*-value significance. We found 143 genes to be upregulated and 333 genes to be downregulated upon pre-miR-326 transfection at the 48 h time point and 114 genes to be upregulated and 64 genes to be downregulated at the 72 h time point (Additional file 6: Tables S5 and Additional file 7: Table S6). In order to select the potential targets of miR-326 from the list of downregulated genes obtained through RNA sequencing, we employed an integrated bioinformatics approach as explained in the schematic (Fig. 5c). A separate analysis of the data from two time points (48 and 72 h) revealed a set of genes that were significantly downregulated (cut-off −0.57 and less in the Log_2_ scale), in either of the time points. We shortlisted the downregulated genes exclusively, as only they could serve as the putative targets of miR-326 (Fig. 5c). Simultaneously, we also extracted the *in silico* gene targets of miR-326 using miRWalk 2.0, an online utility which is a cumulative web-tool of 12 prediction algorithms (Fig. 5c). The intersect of genes predicted *in silico* and the ones downregulated by mir-326 overexpression were taken for further study. The genes were finally shortlisted based on their expression in GBM as compared to control brain. We observed 62 genes to be significantly upregulated in GBM, which satisfied our hypothesis that the genes whose transcript levels were brought down by miR-326, need to be upregulated in GBM in order make a physiological relevance. To further authenticate these genes as the potential targets of miR-326, we carried out a correlation study between their transcript levels and miR-326 expression. We obtained a list of 29 genes that exhibited a significant negative correlation with the miR-326 expression (Fig. 5c) (Additional file 8: Table S7). The list of genes represents the putative targets miR-326. In order to ascribe functional relevance to our gene list of putative miR-326 targets, we carried out an unbiased functional enrichment analysis of this list of 29 genes. The gene ontology and pathway analysis using DAVID unveiled the functional role of miR-326 in gliomagenesis as it depicted a significant enrichment of various terms and pathways related to cell migration like focal adhesion, extracellular matrix-receptor interaction, integrin signalling pathway, cell adhesion mediated signalling, regulation of cell motion (Fig. 6). All these terms essentially explain the phenotype that we observed with miR-326 overexpression in GBM.

**Fig. 7:**
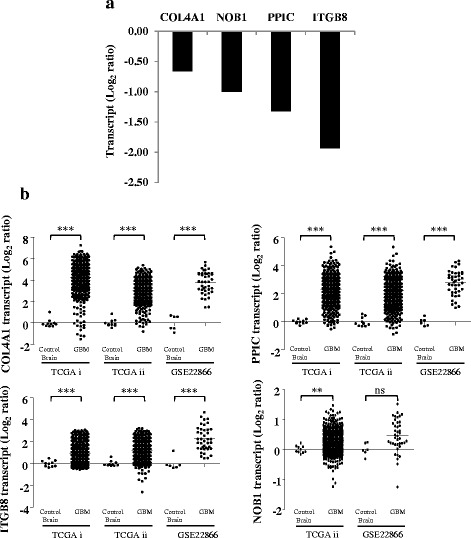
Validation of the putative miR-326 targets. **a**. The column plot depicts the putative targets of miR-326 since they were downregulated upon Pre-miR-4484 transfection, validated through qRT-PCR analysis. **b**. Transcript analysis of the genes downregulated upon miR-326 overexpression. Data derived from TCGA and GSE22866 datasets. TCGA i and TCGA ii refer to Affymetrix and Agilent platform respectively. *p* values were calculated by student’s *t*-test and the symbols are indicated. (ns) not significant; (**) *p* ≤ 0.01 and (***) *p* ≤ 0.001

We further validated some of the genes from the putative target gene list which include COL4A1, NOB1, PPIC, and ITGB8 (Fig. 7a). We also analysed the transcript levels of these genes across other different publicly available datasets (TCGA and GSE22866) and observed that most of them were significantly upregulated in GBM, underscoring the importance of these genes to be the putative targets of miR-326 (Fig. 7b).

## Discussion

miRNAs indeed play a decisive role in tumourigenesis as their misregulation leads to the aberrant expression of their target genes which happen to function as oncogenes or tumour suppressors [21, 22]. In addition to the alterations in the protein coding genes, GBM transcriptome also presents an altered picture of the miRNome [23, 24]. miRNAs can certainly function as oncogenes and tumour suppressors in GBM as well, as has been reported in many studies. There are umpteen studies that have depicted the deregulation of miRNAs in GBM, which in addition to displaying the distorted landscape of miRNome have also proven useful in terms of diagnosis as well as prognosis [25, 26]. Variations in the expression of miRNAs have been associated with almost every facet of tumour biology like, cell proliferation, migration, angiogenesis and chemo-resistance [7, 22].

There is a multitude of ways by which the abundance of miRNAs can get affected. One of the most important and plausible mechanisms that affects the expression of miRNAs, is through transcriptional control. With the aim to elucidate the changes in the miRNome governed by the PI3 kinase pathway, we observed that the abrogation of the PI3 kinase pathway resulted in the alteration of the miRNome of the glioma cells. We identified a set of miRNAs whose expression was elevated upon PI3 kinase pathway inhibition, as well as a cohort of miRNAs whose transcript levels were brought down upon PI3 kinase pathway inhibition. Based upon the concurrence we obtained from their expression values in GBM, we identified miR-326 as one of the PI3 kinase pathway regulated miRNA. Thus, miR-326 is downregulated in GBM owing to the aberrant activity of the PI3 kinase pathway. The downregulation of miR-326 has also been reported previously in few other studies [27, 28]. Ectopic expression of miRNA-326 in glioma and glioma stem cells resulted in the induction of apoptosis and also declined the metabolic activity of the malignant cells [27]. miR-326 has also been shown to effect the viability and invasiveness of glioma cells [29]. In yet another study, miR-326 was identified to regulate stemness by targeting SMO gene, which is an important component of the hedgehog signaling. SMO is upregulated in gliomas and is associated with tumour grade and has prognostic implications as well [30]. miR-326 was also demonstrated as an inhibitor of the Notch pathway and it in turn was also inhibited by Notch [27]. In addition to that, PKM2 was validated as the direct target of miR-326, thereby explaing the metabolic decline in the cells which overexpressed miR-326 [29]. miR-326 along with two other miRNAs (miR-125b and miR-324-5p) were found to regulate the downstream targets of the hedgehog signalling, in particular of Smo and Gli1 [31]. In concurrence with the above observations and reports, we also observed that the reintroduction of miR-326 resulted in a growth suppressive phenotype, affecting the proliferation and migration of glioma cells, thereby indicating that the downregulation of miR-326 by the aberrant PI3 kinase signaling is an important event in the course of gliomagenesis.

In our study, we identified a set of genes which could be the potential targets of miR-326, as they were transcriptionally repressed by miR-326 and many of them happened to be upregulated in GBM as compared to control brain. The gene ontology and pathway analysis of these putative targets of miR-326 revealed a significant enrichment of various terms relevant to migration and invasive phenotype. In fact, we identified a significant enrichment in the integrin signalling, which points to the role of miR-326 in modulating integrin mediated signalling to compromise the migratory phenotype. Some of the putative targets that we narrowed upon like Notch2 and NOB1 have already been proven to be the targets of miR-326 [27, 28]. Among the other targets we demonstrated that miR-326 indeed resulted in the downregulation of COL4A1, NOB, PPIC and ITGB8, possibly explaining the phenotype affected by miR-326 re-expression as these genes can directly affect the processes like cell migration and invasion. COL4A1 encodes a type IV collagen alpha protein and interact with the components of the basement membrane like perlecans, proteoglycans, and laminins. The alteration and further mutilation of the extracellular matrix can definitely have oncogenic propensity. ITGB8, which encodes a protein of integrin beta chain family, mediates cell-cell and cell-extracellular matrix interactions, thereby modulating the extra cellular milieu in favour of tumorigenesis [32]. Thus, in addition to influencing the hedgehog and notch signalling as reported previously, miR-326 can potentially modulate integrin signalling in the course of gliomagenesis.

miR-326 is an intragenic miRNA, possibly a mirtron as its genetic locus is in the first intron of its host gene Arrestin β1 (ARRB1). Both the miRNA and its host genes are located on the same strand (negative) thereby having same orientation. As reported previously, the levels of ARRB1 are also downregulated in glioblastoma [27]. Moreover, we also observed a significant correlation in the expression of miR-326 and ARRB1, possibly suggesting that miR-326 expression was governed by the transcriptional control of ARRB1. This was further corroborated by the fact that the transcript levels of ARRB1 increased upon the inhibition of the PI3 kinase pathway. Our transcription factor analysis revealed various crucial transcription factors like SOX6, INSM1, ZNF300 and ZIC3 to be differentially regulated by the PI3 kinase signaling. These transcription factors significantly correlated with the expression of ARRB1 indicating a possible dependence for its expression. SOX6, INSM1 and ZNF300 were expressed more in the high phospho Akt GBM samples and exhibited a significant negative correlation with ARRB1, thereby indicating that these could function as the potential transcriptional repressors of ARRB1 (Table 1). ZNF300 is a zinc finger protein, expressed mostly in heart, skeletal muscle and brain and exhibits transcription repressor activity [33]. ZNF300 promoter activity was found to be regulated by PU.1 transcription factor in acute promyelocytic leukemia [34]. In yet another report, it was observed that Akt induces the transcriptional activity of PU.1 by modifying it post-translationally through phosphorylation [35]. This hints to the possibility that PI3 kinase pathway can potentially activate ZNF300, which thereby mediates the repression of ARRB1 locus. Moreover, we also observed that, ZNF300 was upregulated in GBM compared to control brain (Table 1). INSM1, yet another zinc-finger transcription factor that could potentially repress ARRB1 locus was also upregulated in GBM compared to control brain (Table 1). It is exclusively expressed in the nervous system and foetal pancreas and functions as transcription repressor in a Cyclin D1 and HDAC dependent fashion [36]. SOX6 was found to specifically expressed either in foetal brain or gliomas, but faintly in the adult brain [37, 38], which again indicates that it might have a role in transformation by repressing various tumour-suppressive targets like the ARRB1 locus. SOX6 was also upregulated in GBM compared to control brain (Table 1). In contrast, ZIC3 was more expressed in the low phospho-Akt GBM samples and exhibited a significant positive correlation with ARRB1 and thus may function as the potential transcriptional activator of ARRB1 (Table 1). The expression of ZIC3 was significantly downregulated in GBM as well, hinting to its function as a positive regulator of ARRB1 (Table 1). Briefly, we identify four transcription factors ZNF300, INSM1, SOX6 and ZIC3 that are regulated by PI3 kinase signalling and can impact the expression of ARRB1-miR-326 locus. Additionally, a study has previously verified that the levels of miR-326 increased when the Notch signalling was abrogated, and there being various reports which furnish substantial evidences of cross-talk between Notch and PI3 kinase [39–41], we speculate that PI3 kinase pathway, by altering the Notch signalling or vice versa, can also result in the transcriptional silencing of the miR-326 locus.

## Conclusions

In summary, our study indicates that the PI3 kinase pathway indeed modulates the expression of miRNAs in a pro-oncogenic manner. We identify various miRNAs whose levels are altered upon silencing of the PI3 kinase pathway. Many of these miRNAs are inversely regulated in glioblastoma. We report here the first evidence that the expression of miR-326 is inhibited by the aberrant PI3 kinase signalling thereby resulting in its downregulation in GBM. In fact, the hyperactivity of PI3 kinase pathway results in the downregulation of an array of tumour suppressor genes and our finding appends this list by one more gene: miR-326. Additionally, miR-326 is significantly downregulated in GBM samples and its reincorporation exhibited tumour suppressive effects.

miR-326 portrays a strong correlation in its expression with its host gene, ARRB1. Later, we even demonstrated that ARRB1 expression is also suppressed by PI3 kinase pathway and is downregulated in GBM as well, suggesting that miR-326 expression is controlled by its host gene regulation which in turn is governed by the PI3 kinase pathway. Our *in silico* analysis of the promoter region of ARRB1 also identifies some of the potential transcription factors which are regulated by PI3 kinase signalling in GBM and can alter the transcription profile of the ARRB1 locus, there regulating miR-326 expression as well. Additionally, we identify various putative targets of miR-326 that are downregulated upon miR-326 overexpression and are also predicted through bioinformatics analysis. Most of these genes are upregulated in GBM and negatively correlate with miR-326 in terms of their abundance, indicating that miR-326 could be mediating its tumour suppressive function by targeting these genes.

## Additional files

Additional file 1: Table S1. List of miRNAs significantly regulated upon PI3 kinase pathway inhibition. (XLSX 19 kb)
Additional file 2: Table S2. List of miRNAs regulated upon PI3 kinase pathway inhibition and their regulation in GBM. (XLSX 12 kb)
Additional file 3: Table S3. Raw expression values along with sample information (Sample ID) corresponding to different scatter plots on separate work sheets. (XLSX 5545 kb)
Additional file 4: Figure S1. miR-326 and ARRB1 share a common promoter upstream to ARRB1. **Figure S2.** Flowchart for the shortlisting of transcription factors regulated by the PI3 kinase pathway. (PPTX 303 kb)
Additional file 5: Table S4. List of transcription factors regulated by PI3 kinase pathway that can potentially regulate ARRB1 locus. (XLSX 13 kb)
Additional file 6: Table S5. Gene regulated by miR-326 overexpression at 48 h interval. (XLSX 44 kb)
Additional file 7: Table S6. Gene regulated by miR-326 overexpression at 72 h interval. (XLSX 22 kb)
Additional file 8: Table S7. List of genes downregulated upon miR-326 overexpression, upregulated in GBM and bearing binding sites for miR-326. (XLSX 12 kb)

## Abbreviations

ANOVA: Analysis of variance.
ARRB1: Arrestin beta 1
ECACC: European Collection of Authenticated Cell Cultures
GBM: Glioblastoma
LNA: Locked nucleic acid
miR: microRNA
MTT: [3-(4,5-Dimethylthiazol-2-yl)-2,5-Diphenyltetrazolium Bromide]
Pre-miR: Precursor miRNA
REMBRANDT: Repository of molecular brain Neoplasia Data
RTK: Receptor tyrosine kinase
TCGA: The cancer genome atlas
