# Supplementary material for: PI3 kinase pathway regulated miRNome in glioblastoma: identification of miR-326 as a tumour suppressor miRNA
Source: Mol Cancer. 2016 Nov 21;15:74. doi: 10.1186/s12943-016-0557-8 (PMC5117574; doi:10.1186/s12943-016-0557-8)
Supplement: Additional file 4: Figure S1. — miR-326 and ARRB1 share a common promoter upstream to ARRB1. Figure S2. Flowchart for the shortlisting of transcription factors regulated by the PI3 kinase pathway. (PPTX 303 kb) [file 12943_2016_557_MOESM4_ESM.pptx]

## Slide 1
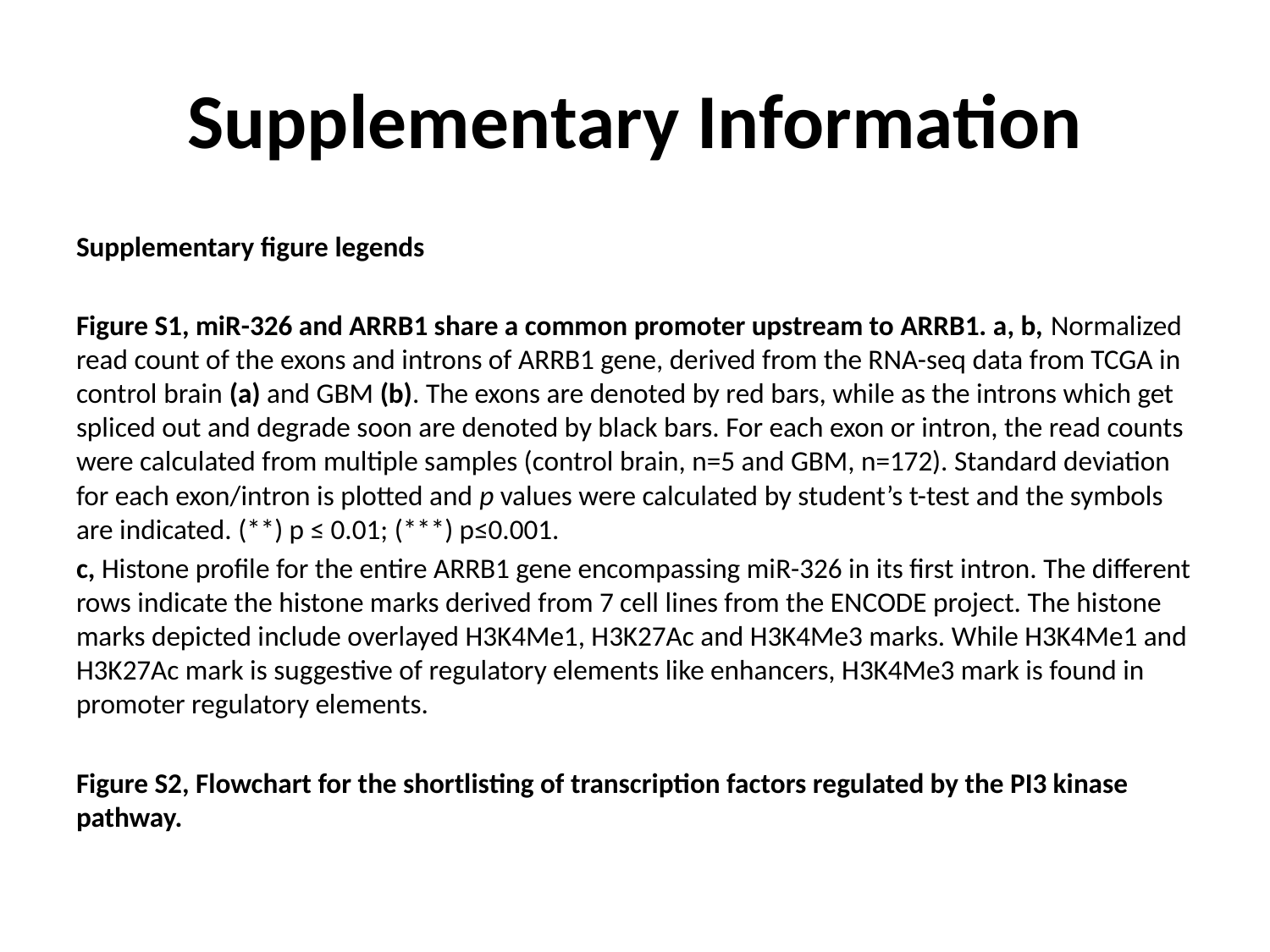

# Supplementary Information
Supplementary figure legends
Figure S1, miR-326 and ARRB1 share a common promoter upstream to ARRB1. a, b, Normalized read count of the exons and introns of ARRB1 gene, derived from the RNA-seq data from TCGA in control brain (a) and GBM (b). The exons are denoted by red bars, while as the introns which get spliced out and degrade soon are denoted by black bars. For each exon or intron, the read counts were calculated from multiple samples (control brain, n=5 and GBM, n=172). Standard deviation for each exon/intron is plotted and p values were calculated by student’s t-test and the symbols are indicated. (**) p ≤ 0.01; (***) p≤0.001.
c, Histone profile for the entire ARRB1 gene encompassing miR-326 in its first intron. The different rows indicate the histone marks derived from 7 cell lines from the ENCODE project. The histone marks depicted include overlayed H3K4Me1, H3K27Ac and H3K4Me3 marks. While H3K4Me1 and H3K27Ac mark is suggestive of regulatory elements like enhancers, H3K4Me3 mark is found in promoter regulatory elements.
Figure S2, Flowchart for the shortlisting of transcription factors regulated by the PI3 kinase pathway.

## Slide 2
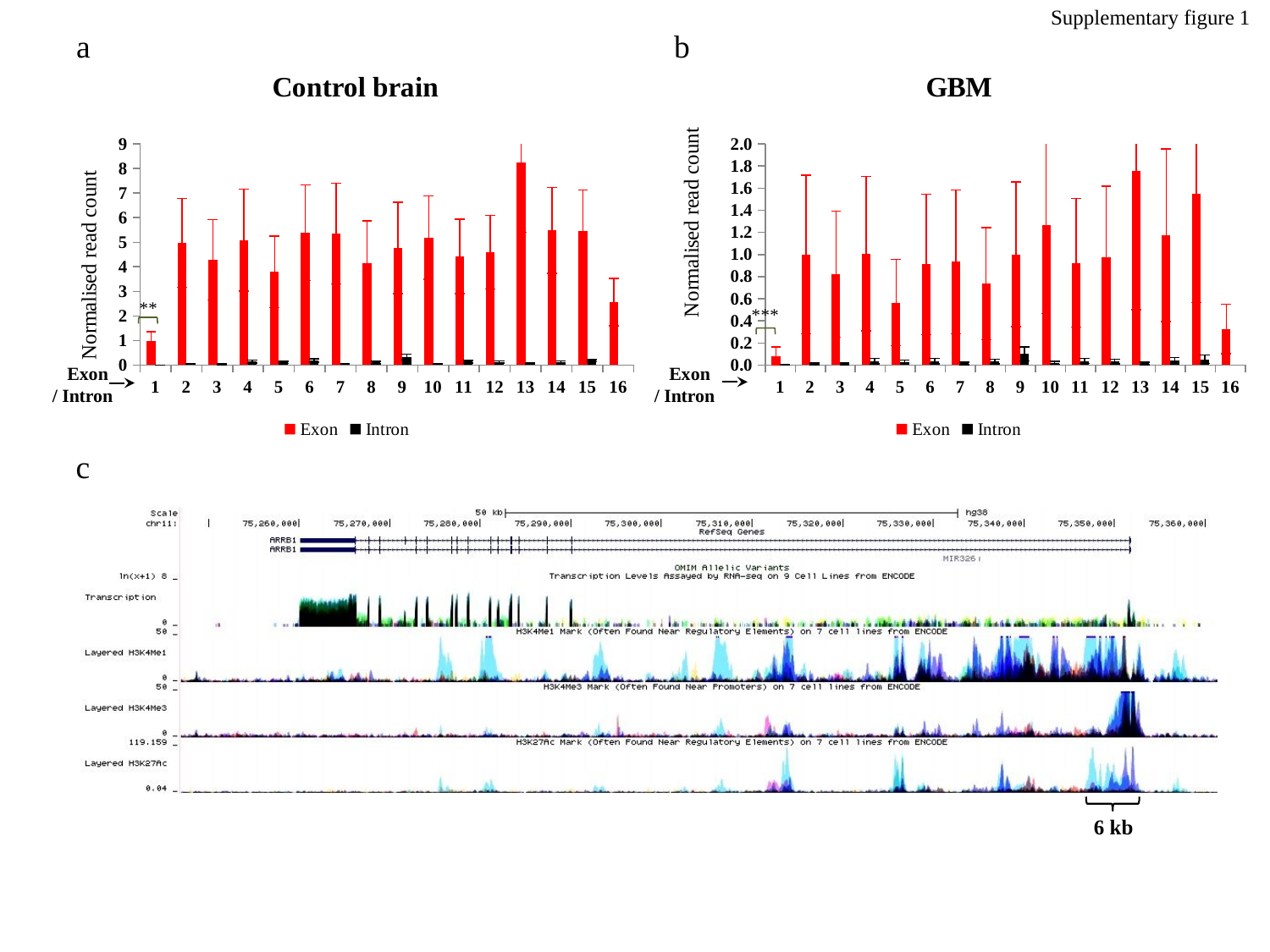

Supplementary figure 1
a
b
[unsupported chart]
Exon
/ Intron
### Chart: GBM
| Category | | |
|---|---|---|
Exon
/ Intron
c
6 kb

## Slide 3
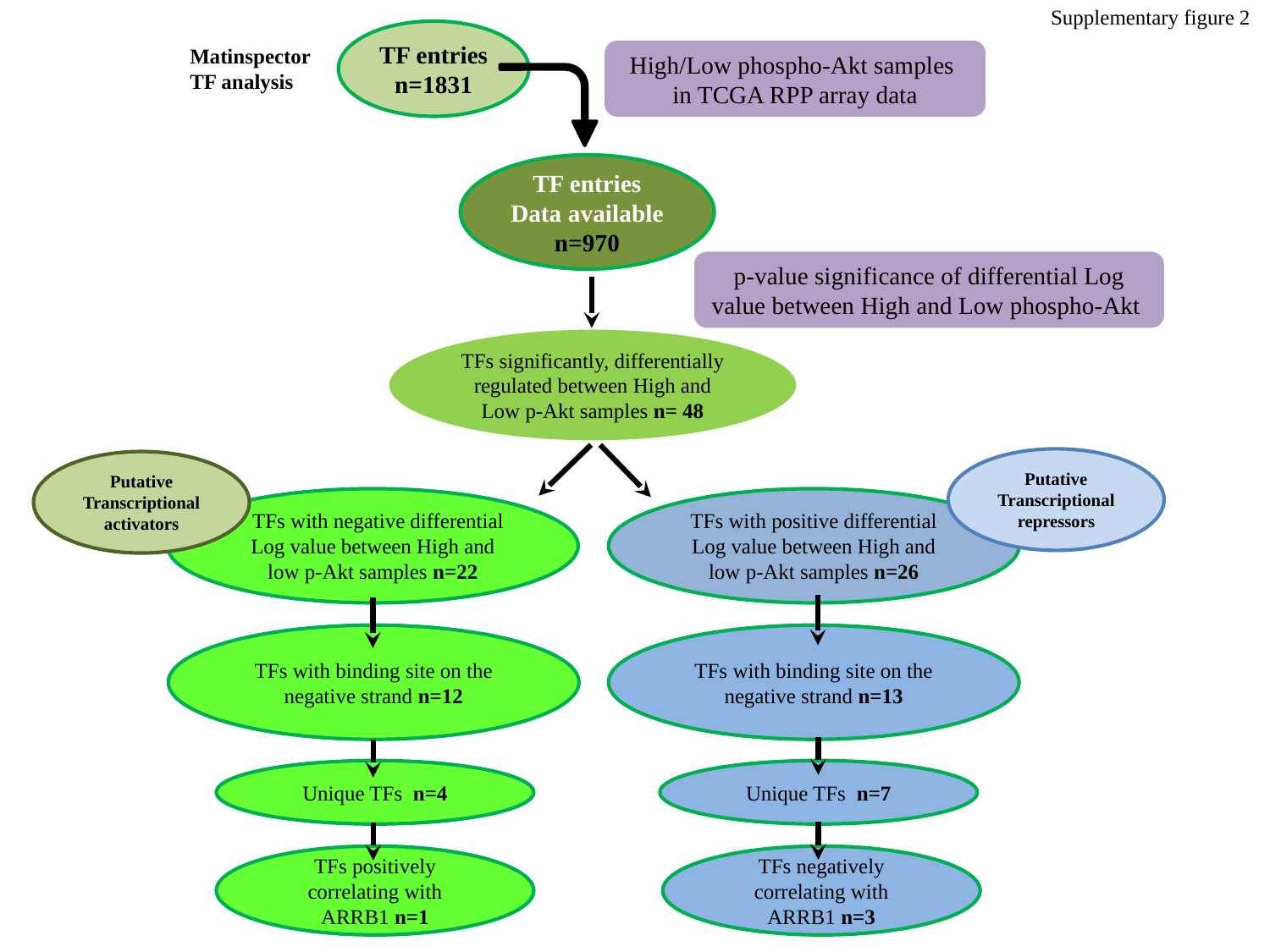

Supplementary figure 2
TF entries n=1831
Matinspector
TF analysis
High/Low phospho-Akt samples
in TCGA RPP array data
TF entries
Data available
n=970
p-value significance of differential Log value between High and Low phospho-Akt
TFs significantly, differentially regulated between High and Low p-Akt samples n= 48
Putative
Transcriptional repressors
Putative Transcriptional activators
 TFs with negative differential Log value between High and low p-Akt samples n=22
TFs with positive differential Log value between High and low p-Akt samples n=26
TFs with binding site on the negative strand n=12
TFs with binding site on the negative strand n=13
Unique TFs n=4
Unique TFs n=7
TFs positively correlating with ARRB1 n=1
TFs negatively correlating with ARRB1 n=3
